# Supplementary material for: Blood test dynamics in hospitalized COVID-19 patients: Potential utility of D-dimer for pulmonary embolism diagnosis
Source: PLoS One. 2020 Dec 28;15(12):e0243533. doi: 10.1371/journal.pone.0243533 (PMC7769556; doi:10.1371/journal.pone.0243533)
Supplement: S1 Table — (DOCX) [file pone.0243533.s002.docx]

**SUPPLEMENTARY MATERIAL**

**Article: Blood test dynamics in hospitalized patients with COVID‑19: potential utility of D-dimer for pulmonary embolism diagnosis**

| **Supplementary Material Table 1. Characteristics of pulmonary embolism (PE)** | |
| --- | --- |
|  | **PE**  **N= 29** |
| **Days from onset of COVID‑19 symptoms to PE diagnosis (mean ±**  **SD )** | 20.0 (SD  8.6) |
| **CT radiological COVID‑19 pneumonia status at PE diagnosis**  **Worse**  **No change** | 10 (34%)  13 (45%) |
| **Bilateral pulmonary embolism, n (%)** | 15 (52%) |
| **Vessel involved, n (%)** |  |
| Subsegmental | 3 (10%) |
| Segmental | 10 (34%) |
| Lobar | 9 (31%) |
| Main artery | 7 (24%) |
| **Pulmonary infarction, n (%)** | 4 (14%) |
| **Pleural effusion, n (%)** | 8 (28%) |
| **Pericardial effusion, n (%)** | 2 (7%) |
| **Pneumothorax / pneumomediastinum** | 2 (7%) |
| **RV / LV ratio > 1, n (%)** | 3 (10%) |
| **Thrombus location in the arterial vessel, n (%)** |  |
| Central | 13 (45%) |
| Peripheral | 5 (17%) |
| Complete occlusion | 10 (34%) |
| **sPESI score** | 1.43 (SD  0.9) |
| **PE and COVID‑19 lung involvement anatomically related, n (%)** | 7 (24%) |
| **Lower limbs deep venous thrombosis/ Doppler US** | 6 (21%) / 6 |
| Right side | 6 (21%) |
| sPESI, simplified Pulmonary Embolism Severity Index; RV /LV ratio, right to left ventricular; US, ultrasound. | |
