# Supplementary material for: Blood test dynamics in hospitalized COVID-19 patients: Potential utility of D-dimer for pulmonary embolism diagnosis
Source: PLoS One. 2020 Dec 28;15(12):e0243533. doi: 10.1371/journal.pone.0243533 (PMC7769556; doi:10.1371/journal.pone.0243533)
Supplement: S2 Table — (DOCX) [file pone.0243533.s003.docx]

| **Supplementary Material Table 2. Blood test results during hospitalization across different time periods from COVID‑19 onset of symptoms according to the occurrence of pulmonary embolism (PE)** | | | | | | | | | | | | |
| --- | --- | --- | --- | --- | --- | --- | --- | --- | --- | --- | --- | --- |
| **Blood test** | **Weeks from COVID‑19 symptoms onset** | | | | | | | | | | | |
|  | **Week 1** | | **Week 2** | | **Week 3** | | **Week 4** | | **Week 5** | | **Week 6** | |
|  | **PE** | **No‑PE** | **PE** | **No‑PE** | **PE** | **No‑PE** | **PE** | **No‑PE** | **PE** | **No‑PE** | **PE** | **No‑PE** |
| **Urea, mmol/L** | 5.8  SD 1.7 | 7.9  SD 5.1 | 7.4  SD 3.1 | 8.9  SD 4.6 | 9.5  SD 4.7 | 9.8  SD 4.6 | 9.7  (6.8‑11.4) | 8.3  (6.4‑12.3) | 9.8  SD 5.3 | 11.7  SD 7.1 | 6.3  SD 3.2 | 11.1  SD 7.7 |
| **Creatinine, µmol/L** | 75  (59‑83) | 83.2  (62.8‑110.9 | 78  (57.3‑101) | 83.4  (67.7‑110) | 74.7  (71.2‑95) | 78.2  (61‑99.6) | 72.4  (59.3‑96.8) | 78  (53.3‑127) | 82  (59.7‑96.3) | 69.3  (47.8‑130.5) | 64.8  (53.6‑72.7) | 52  (41.9‑97.8) |
| **FG, mL/min** | 83.0  SD 20.7 | 72.1  SD 28.0 | 78.6  SD 21.6 | 67.6  SD 25.4 | 78.1  (69.6‑94.3) | 82.4  (60.3‑95) | 82  (74.9‑95) | 84.3  (57.1‑95) | 80.3  SD 14.9 | 73.4  SD 27.8 | 95  (90.3‑95) | 95  (74.8‑95) |
| **LDH, U/L** | 385.3  SD 173.3 | 337.8  SD 151.9 | 447.7  SD 203.9 | 379.1  SD 137.2 | 442.9  SD 256.1 | 363.3  SD 140.3 | 399.1  SD 217.8 | 399.7  SD 220.8 | 399.9  SD 251.6 | 402.8  SD 204.0 | 312.7  SD 152.4 | 342.4  SD 133.7 |
| **CK, U/L** | 53.5  (24.2- 100.5) | 64.5  (45.2- 100.5) | 189.2  (41.1- 267.6) | 76.9  (40.7- 199.6) | 41  (34.3‑90.8) | 63.6  (33.2‑109) | 41.4  (33.5‑180) | 37.2  (24.2‑  127.6) | 62.6  (56.1‑173.4) | 44.3  (28.7‑292.9) | 26.1  (20‑171.4) | 25  (19‑60.6) |
| **Troponin, ng/L** | **11.0**  **SD 5.8*** | **22.3**  **SD 12.3*** | 10.4  (7.8‑29.7) | 17.1  (12.4‑24.7) | **13.3**  **(9.8‑15.3)*** | **19**  **(12.6‑ 28.6)*** | 19.6  (10.9‑26.1) | 21  (11.5‑47.1) | 26.6  (13.8‑62.1) | 24.6  (19.4‑45.8) | 23.2  (11.1‑81.7) | 36.9  (14.9‑43.4) |
| **ALT, U/L** | **30.8**  **(19.8‑ 55.3)*** | **18**  **(11.9‑ 23.8)*** | 37.6  (26.8‑52.6) | 36.9  (18.2‑55) | 55.2  (38.6‑104.1) | 42.4  (28.1‑67.9) | 66.2  SD 41.9 | 69.2  SD 52.7 | 62.4  (41.5‑100.4) | 36.9  (21.2‑62) | 34.4  SD 15.1 | 39.5  SD 25.7 |
| **AST, U/L** | 36.1  SD 14.5 | 40.2  SD 30.3 | 47.9  SD 29.1 | 51.2  SD 28.9 | 48.6  SD 24.5 | 44.0  SD 26.0 | 36.5  (21.5‑56.6) | 35.9  (26.8‑54.8) | 39.8  SD 34.2 | 52.1  SD 53.2 | 28.1  SD 15.7 | 31.3  SD 20.6 |
| **Albumin, g/L** | 36.3  SD 3.2 | 34.8  SD 4.7 | 35.4  SD 3.7 | 32.1  SD 4.2 | 31.7  SD 4.5 | 31.1  SD 4.8 | 29.5  SD 4.8 | 30.5  SD 5.5 | 29.6  SD 5.7 | 29.6  SD 5.6 | 31.0  SD 5.9 | 31.1  SD 4.9 |
| **Ferritin, µg/L** | 563.5  SD 190.2 | 690.7  SD 467.9 | 1870.6  SD 1149.4 | 1410.8  SD 1077.8 | 1449.8  SD 810.5 | 1391.5  SD 1061.3 | 1432.4  SD 906.4 | 1195.5  SD 789.9 | 1673.5  (1118.9  ‑2661.7) | 975.9  (434.1‑  1614.5) | 1329.4  SD 1230.8 | 937.8  SD 746.1 |
| **IL‑6, ng/L** | 0 | 18.2  SD 3.7 | 104.4  (61.6‑  1854.8) | 146.5  (38.9‑  848.6) | 1056.5  SD 1045.9 | 1078.8  SD1664.3 | 276.7  SD 291.9 | 303.6  SD 324.1 | 136.4  SD 134.3 | 623.6  SD 847.4 | 63.7  SD 49.9 | 72.5  SD 53.2 |
| **C‑reactive protein, mg/L** | 114.9  SD 114.2 | 74.6  SD 51.2 | 165.4  SD 116.9 | 116.9  SD 80.9 | 80  (24.3‑100.9) | 50.3  (11.6‑103) | 98.8  (18.3‑98.8) | 21.1  (2.9‑88.8) | 102.4  SD 101.2 | 70.7  SD 60.6 | 62.0  SD 68.2 | 50.0  SD 58.5 |
| **Procalcitonin, µg/L** | 0.1 | 4.6  SD 7.7 | 0.33  (0.1‑0.39) | 0.26  (0.15‑0.82) | 0.14  (0.08‑0.39) | 0.17  (0.1‑0.63) | 0.27  (0.05‑0.75) | 0.2  (0.06‑1.2) | 0.27  (0.06‑0.87) | 0.3  (0.1‑3.3) | 0.3  SD 0.4 | 0.7  SD 1.1 |
| **Supplementary Material Table 2 (cont.). Blood test results during hospitalization across different time periods from COVID‑19 onset of symptoms according to the occurrence of pulmonary embolism (PE)** | | | | | | | | | | | | |
|  | **Weeks from COVID‑19 symptoms onset** | | | | | | | | | | | |
|  | **Week 1** | | **Week 2** | | **Week 3** | | **Week 4** | | **Week 5** | | **Week 6** | |
|  | **PE** | **No‑PE** | **PE** | **No‑PE** | **PE** | **No‑PE** | **PE** | **No‑PE** | **PE** | **No‑PE** | **PE** | **No‑PE** |
| **Triglycerides, mmol/L** | 2.1  SD 0.1 | 1.8  SD 0.3 | 2.6  SD 1.7 | 2.5  SD 1.2 | 2.9  SD 4.5 | 2.7  SD 1.3 | 2.3  SD 0.9 | 2.6  SD 1.1 | 2.4  SD 0.9 | 2.5  SD 1.5 | 2.1  SD 0.5 | 2.2  SD 0.9 |
| **Hemoglobin, g/L** | **140.5**  **SD 22.5*** | **124.9**  **SD 21.8*** | 129.2  SD 17.4 | 121.3  SD 16.9 | **131.1**  **SD 20.8*** | **117.1**  **SD 20.9*** | 118.8  SD 19.3 | 111.3  SD 22.1 | 106.3  SD 19.3 | 103.5  SD 23.7 | 100.3  SD 16.5 | 98.6  SD 20.9 |
| **Platelets, ×10^9^** | 212.1  SD 110.2 | 199.6  SD 107.1 | 261.1  SD 88.9 | 262.5  SD 131.0 | 303.2  SD 105.7 | 321.9  SD 123.9 | 267.2  SD 116.9 | 264.0  SD 111.6 | 247.6  SD 84.5 | 247.3  SD 143.3 | 307.8  SD 114.7 | 251.9  SD 112.5 |
| **Leukocytes, ×10^9^** | 8.4  SD 5.1 | 6.6  SD 3.6 | 9.3  SD 3.4 | 8.3  SD 4.5 | 11.5  SD 5.4 | 9.9  SD 4.6 | 11.9  SD 5.6 | 10.7  SD 5.7 | 10  (8.1‑14.1) | 11.4  (7.7‑13.6) | 14.0  SD 13.2 | 10.2  SD 4.7 |
| **Neutrophils (T), ×10^9^** | 6.7  SD 4.8 | 4.9  SD 3.3 | 4.7  SD 3.5 | 6.8  SD 4.2 | 9.6  SD 4.5 | 8.1  SD 4.4 | 11.4  SD 5.9 | 8.6  SD 5.7 | 12.1  SD 11.6 | 8.9  SD 4.8 | 6.7  SD 1.4 | 7.8  SD 4.7 |
| **Neutrophils (%) ×10^9^** | 77.1  SD 8.6 | 72.3  SD 12.5 | 81.0  SD 8.4 | 79.4  SD 9.6 | 81.6  SD 9.9 | 78.5  SD 11.3 | 78.7  SD 16.8 | 75.9  SD 13.6 | 78.6  SD 11.5 | 77.6  SD 12.2 | 72.2  SD 7.3 | 71.2  SD 11.8 |
| **Lymphocytes (T), ×10^9^** | 1.0  SD 0.5 | 1.0  SD 0.8 | 1.1  SD 0.6 | 0.8  SD 0.4 | 2.4  SD 6.3 | 1.1  SD 0.7 | 1.3  SD 0.7 | 1.2  SD 0.8 | 1.5  SD 0.9 | 1.2  SD 0.7 | 1.8  SD 0.9 | 1.4  SD 0.6 |
| **Lymphocytes (%)** | 14.9  SD 6.2 | 16.9  SD 9.6 | 12.3  SD 6.1 | 12.2  SD 6.5 | 9.4  (4.7‑14.6) | 9.9  (6.6‑18.3) | 13.2  SD 11.9 | 14.3  SD 10.1 | 12.6  SD 9.7 | 12.7  SD 8.4 | 16.6  SD 7.1 | 17.2  SD 10.2 |
| **Prothrombin time** | 1.09  SD 0.06 | 1.14  SD 0.21 | 1.26  (1.1‑1.4) | 1.2  (1.1‑1.2) | 1.14  (1.1‑1.32) | 1.1  (1.1‑1.2) | **1.27**  **(1.11‑1.43)*** | **1.11**  **(1‑1.2)*** | 1.25  (1.1‑1.4) | 1.1  (1.1‑1.2) | 1.12  SD 0.10 | 1.12  SD 0.17 |
| **Activated partial thromboplastin time** | 0.99  SD 0.12 | 1.03  SD 0.13 | 1.07  SD 0.27 | 1.07  SD 0.13 | 1.05  SD 0.15 | 1.04  SD 0.16 | 1.08  SD 0.12 | 1.03  SD 0.13 | 1.14  SD 0.12 | 1.09  SD 0.24 | **1.20**  **SD 0.11*** | **1.03**  **SD 0.08*** |
| **Fibrinogen** | 7.6  SD 1.6 | 6.6  SD 1.9 | 8.6  (6.8‑9) | 7.9  (6.4‑9) | 6.1  SD 1.9 | 6.2  SD 1.9 | 6.1  SD 2.5 | 5.7  SD 2.3 | 7.1  SD 2.1 | 6.7  SD 2.1 | 6.3  SD 1.7 | 6.5  SD 1.7 |
| **D‑dimer** | 389.8  (200-1794.8) | 742  (340.2-2602.8) | **2010.7**  **(770.1-11209)*** | **626**  **(374-2382.3)*** | **3893.1**  **(1388.2-6694)*** | **1184.4**  **(461.8-2447.8)*** | **2736.3**  **(1202.1-8514.1)*** | **1129.1**  **(542.5-2834.6)*** | 2376.9  SD 1874.7 | 1667.2  SD 1570.0 | 1292.7  SD 1141.9 | 1045.8  SD 1063.9 |
| Results are shown as means with standard deviations (SD) or median with interquartile range [IQR]. Results in bold with an asterisk indicate statistically significant differences. | | | | | | | | | | | | |
