# Supplementary material for: Blood test dynamics in hospitalized COVID-19 patients: Potential utility of D-dimer for pulmonary embolism diagnosis
Source: PLoS One. 2020 Dec 28;15(12):e0243533. doi: 10.1371/journal.pone.0243533 (PMC7769556; doi:10.1371/journal.pone.0243533)
Supplement: S3 Table — (DOCX) [file pone.0243533.s004.docx]

| **Supplementary Material Table 3. D‑dimer results during hospitalization across different time periods from COVID‑19 onset of symptoms according to the occurrence of pulmonary embolism (PE)** | | | | | | | | | | | | |
| --- | --- | --- | --- | --- | --- | --- | --- | --- | --- | --- | --- | --- |
| **D‑Dimer cut‑off points (µg/L)** | **Weeks from COVID‑19 symptoms onset** | | | | | | | | | | | |
|  | **Week 1** | | **Week 2** | | **Week 3** | | **Week 4** | | **Week 5** | | **Week 6** | |
|  | **PE** | **No‑PE** | **PE** | **No‑PE** | **PE** | **No‑PE** | **PE** | **No‑PE** | **PE** | **No‑PE** | **PE** | **No‑PE** |
| **Reference normal range** | | | | | | | | | | | | |
| **≤250** | 4/10 (40%) | 2/22 (9%) | 0/18 (0%) | 5/51 (10%) | 0/0  (0%) | 8/54 (15%) | 0/12 (0%) | 4/40 (10%) | 1/7 (14%) | 0/0  (0%) | 1/5 (20%) | 0/0  (0%) |
| **>250** | 6/10 (60%) | 20/22 (91%) | 18/18 (100%) | 46/51 (90%) | 16/16 (100%) | 46/54 (85%) | 12/12 (100%) | 36/40 (90%) | 6/7 (86%) | 25/25 (100%) | 4/5 (80%) | 17/17 (100%) |
| **Age‑adjusted cut-off points** | | | | | | | | | | | | |
| **NEGATIVE** | 4/10 (40%) | 11/22 (50%) | **2/18 * (11%)** | **27/51 (53%)** | **1/16 * (6%)** | **19/54 (35%)** | **0/0 ***  **(0%)** | **15/40 (38%)** | 1/7 (14%) | 5/25 (20%) | 1/5 (20%) | 7/17 (41%) |
| **POSITIVE** | 6/10 (60%) | 11/22 (50%) | **16/18 * (89%)** | **24/51 (47%)** | **15/16 * (94%)** | **35/54 (65%)** | **12/12 * (100%)** | **25/40 (62%)** | 6/7 (86%) | 20/25 (80%) | 4/5 (80%) | 10/17 (59%) |
| **Optimal cut‑off points according to Youden’s *J* statistic** | | | | | | | | | | | | |
| **≤632** |  |  | 11/18 (61%) | 38/51 (75%) |  |  |  |  |  |  |  |  |
| **>632** |  |  | 7/18 (39%) | 13/51 (26%) |  |  |  |  |  |  |  |  |
| **≤2036** |  |  |  |  | **5/16* (31%)** | **40/54 (74%)** |  |  |  |  |  |  |
| **>2036** |  |  |  |  | **11/16* (69%)** | **14/54 (26%)** |  |  |  |  |  |  |
| **≤2271** |  |  |  |  |  |  | **4/12* (33%)** | **30/40 (75%)** |  |  |  |  |
| **>2271** |  |  |  |  |  |  | **8/12* (67%)** | **10/40 (25%)** |  |  |  |  |
| Results in bold with an asterisk indicate statistically significant differences | | | | | | | | | | | | |
